# Evaporation of droplets in a Champagne wine aerosol

**DOI:** 10.1038/srep25148

**Published:** 2016-04-29

**Authors:** Elisabeth Ghabache, Gérard Liger-Belair, Arnaud Antkowiak, Thomas Séon

**Affiliations:** 1 Université Pierre et Marie Curie and Centre National de la Recherche Scientifique, Unité Mixte de Recherche 7190, Institut Jean Le Rond d’Alembert, 4 Place Jussieu, F-75005 Paris, France; 2 Equipe Effervescence (GSMA), UMR CNRS 7331, Université de Reims Champagne-Ardenne, BP 1039, 51687 Reims, France

## Abstract

In a single glass of champagne about a million bubbles nucleate on the wall and rise towards the surface. When these bubbles reach the surface and rupture, they project a multitude of tiny droplets in the form of a particular aerosol holding a concentrate of wine aromas. Based on the model experiment of a single bubble bursting in idealized champagnes, the key features of the champagne aerosol are identified. In particular, we show that film drops, critical in sea spray for example, are here nonexistent. We then demonstrate that compared to a still wine, champagne fizz drastically enhances the transfer of liquid into the atmosphere. There, conditions on bubble radius and wine viscosity that optimize aerosol evaporation are provided. These results pave the way towards the fine tuning of flavor release during sparkling wine tasting, a major issue for the sparkling wine industry.

On a large scale, sea spray, which transports dissolved gases, salts, surfactants, and biological materials to the atmosphere, is largely attributed to aerosols produced by an estimated 10^18^ to 10^20^ bubbles that burst every second across the oceans^1^,^2^,^3^,^4^,^5^,^6^. In particular, the sea-air exchange of surfactant materials has been well described as these aerosols are enriched with surface active agents^7^,^8^,^9^. Indeed, amphiphilic properties of surfactants lead them to the sea surface and consequently to the sea spray.

On a smaller scale the situation found in glasses of champagne, sparkling wines, and fizzy beverages in general, is finally very similar. As they are poured into a glass, the myriad of ascending bubbles collapse and therefore radiate a multitude of tiny droplets above the free surface, in the form of very characteristic and refreshing aerosols^10^ (Fig. 1). Indeed, it is now generally recognized that bubbles bursting at a liquid surface eject two kinds of droplets^11^: (*i*) small droplets, called *film drops*, formed as the film of the emerged bubble-cap disintegrates^12^,^13^,^14^, and (*ii*) bigger *jet drops*, formed as the so-called “Worthington jet”, driven by the collapse of the unstable immersed cavity^15^,^16^,^17^, ruptures (Fig. 2)^18^,^19^.

Based on a phenomenological analogy between the fizz of the ocean and the fizz in Champagne wines, ultrahigh resolution mass spectrometry was used in order to analyze the droplets released by bubbles bursting in champagne^20^. It was found that this aerosol was indeed considerably enriched, compared with the champagne bulk, with chemical compounds showing both surface activity and organoleptic interest. It actually holds the organoleptic “essence” of champagne. This recent discovery supports the idea that rising and collapsing bubbles act as a continuous paternoster lift for aromas above every glass of champagne or any flavored carbonated beverage. We may now wonder how these aromas included in the aerosol will spread in the air and whether we can optimize this diffusion or not.

In this paper, based on the model experiment of a single bubble bursting in idealized champagnes, we characterize the entire drop dynamics, from ejection to evaporation, and we address some of the crucial questions suggested above on champagne aerosol. We show that film drops, usually critical in bursting bubble aerosol, are here nonexistent. We exhibit that hydro-alcoholic solution is an excellent idealized champagne in terms of bubble bursting and jet dynamics. We also demonstrate that champagne fizz really enhances the transfer of liquid into the atmosphere in comparison to a non-sparkling wine. In this context, the conditions on the aerosol generation, that optimize aroma diffusion, are provided. We show that weakly viscous wine and large bubbles improve aerosol evaporation. This work aims at shedding light and providing first quantitative results on champagne aroma diffusion. Furthermore, it could have widespread implications ranging from ocean-atmosphere exchange to the earthy smell, known as ‘petrichor’, present after a rain shower on a hot day^21^.

## Results

### Film drops

As introduced above, bursting bubble usually leads to drop production through two different mechanisms: *film drops* through film retraction and *jet drops* when jet ruptures. Figure 3(a) shows a sequence of a bubble lying at the free surface of water, after a few seconds the cap film punctures and retracts. During retraction the rim suffers an inertial destabilization of a Rayleigh–Taylor type, which leads to the formation of ligaments. Ligaments are then stretched out by centrifugation, producing disjointed droplets by a Plateau–Rayleigh destabilization^14^. These are the so-called *film drops* that we observe on the two last pictures of the sequence (a). In water and for the bubble radius considered here (*R*_*b*_ = 1.7 mm) film rupture should generate thirty-four drops of average radius seventeen microns^14^. But this only applies to water. In particular, Fig. 3(b) presents the same film retraction sequence in champagne, and in this case we observe *no film drops*. The retraction velocity appears lower in champagne suggesting that destabilization of the rim cannot develop before the film has disappeared^22^.

This unexpected result might have various causes as champagne is different from water in many aspects. It is first more viscous (1.6 mPa.s at room temperature) and has a lower surface tension (48 mN.m^−1^). Gradients of surface tension, inherent to hydro-alcoholic solution^23^, can lead to a film thickening by Marangoni effect before it bursts. The reason of this disappearance of film drops is beyond the scope of this paper but definitively constitutes a fascinating problem which will be addressed. It actually makes champagne aerosol very different from sea spray and an interesting model aerosol only populated by jet drops.

### Jet drops

**Figure 2:**
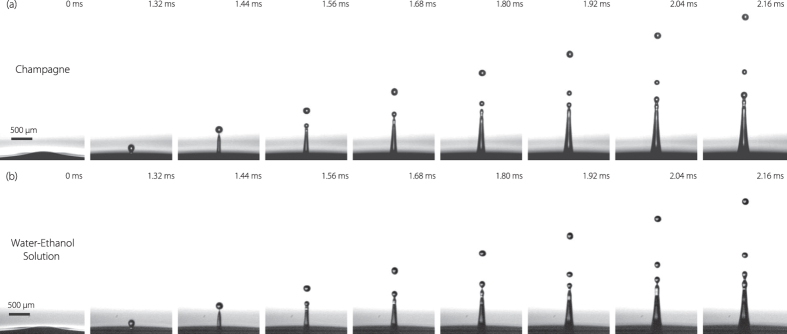
Experimental time sequences of the typical jetting events following a bubble bursting at a free surface of (**a**) a Champagne wine at 20 °C (viscosity *μ* = 1.6 mPa.s, surface tension *γ* = 48 mN.m^−1^ and density *ρ* = 992 kg.m^−3^) and (**b**) a water-ethanol solution (*resp.* 89,5% and 10,5% of total weight) with the same properties. Space scale is on each sequence and times are shown on the snapshots. Bubble radius *R*_*b*_ is almost the same for the two sequences: (**a**) *R*_*b*_ = 817 *μ*m and (**b**) *R*_*b*_ = 830 *μ*m.

Figure 2(a) displays a sequence of a jetting event following a bubble bursting at the free surface of a Champagne wine. The first image shows the static bubble, then the film separating the bubble from the atmosphere drains and bursts leaving an unstable opened cavity. This (sub)millimetric cavity relaxes due to capillary forces and gives rise to the high speed vertical jet shooting out above the free surface as observed on the sequence. The jet then fragments into droplets, generating an aerosol of a few jet drops, the only constituent of a champagne aerosol.

### Idealized Champagne wine

**Figure 3:**
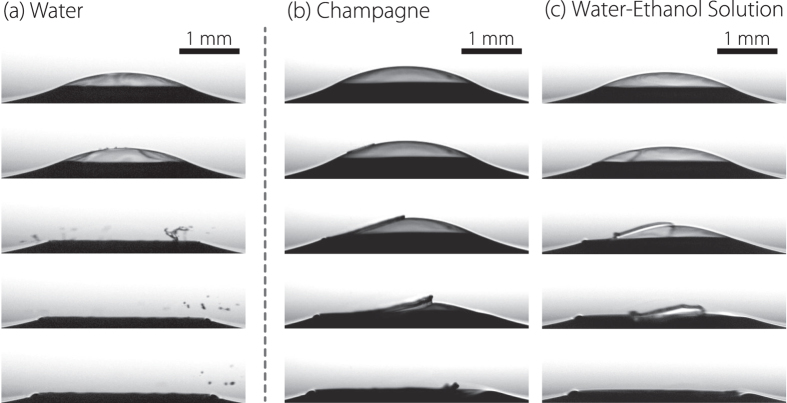
Experimental time sequences of a bubble of radius *R*_*b*_ = 1.7 mm bursting in: (**a**) demineralized water, (**b**) champagne at 20 °C and (**c**) a water-ethanol solution with the same properties. Liquid properties are the same as in Fig. 2. Space scale is on each sequence. The time between each image is: (**a**) Δt = 77 *μ*s, (**b**) Δt = 83 *μ*s and (**c**) Δt = 77 *μ*s.

In the aim of establishing an idealized champagne for our study, the same bursting bubble experiment has been realized in a simple water-ethanol solution (*resp.* 89, 5% and 10, 5% of total weight), allowing us to mimic the liquid properties of a standard Champagne wine at 20 °C: viscosity *μ* = 1.6 mPa.s and surface tension *γ* = 48 mN.m^−1 ^10. Figures 2(b) and 3(c) respectively present the jetting event and the film retraction sequence bursting in our hydro-alcoholic solution for a bubble of the same size as Figs 2(a) and 3(b). The sequences are very close to each other. In particular no film drops are produced and the jet drops look very similar. Therefore, both in terms of the rim retraction and the jet dynamics, a champagne at room temperature is qualitatively perfectly mimicked by a water-ethanol solution with the same properties. As a consequence, the role of surfactants - always present in champagne - is negligible in the bubble bursting dynamics and the drop ejection. These surprising qualitative results will be quantitatively confirmed in the following.

Champagne is a complex hydro-alcoholic solution holding tens of aromatic compounds, and a small amount of surface-active macromolecules with a concentration of only a few milligrams per liter^24^. Such a small amount of surface-active materials in champagne was actually found to have only little effect on ascending bubble dynamics^24^. It also has almost no effect on equilibrium surface tension as, our hydro-alcoholic solution has the same surface tension as a real champagne (see Table 1), with a very close relative amount of ethanol: 13% of total volume in our solution and 12.5% in champagne. All these arguments support the idea that surfactants do not affect the dynamics of bursting bubbles in a real champagne. Consequently, we believe that it is reasonable not to take surfactants into account in a first model of evaporation of jet droplets above a champagne surface. Nevertheless, it makes no doubt that the fine study of such influence should be carried out in further studies.

Furthermore, champagne is always served at low temperature, which leads to a higher liquid viscosity. Glycerol, a water-soluble viscous liquid, can therefore be added in the mixtures to tune their viscosity and mimic this temperature effect. Consequently, the major part of the experiments will be carried out with three mixtures of water, ethanol and glycerol - solutions (*i*)–(*iii*) - with *γ* = 48 mN.m^−1^ and respectively *μ* = 1.6, 2.6 and 3.6 m.Pa.s (see Table 1). These three solutions reproduce the liquid properties of a usual champagne at three different temperatures, namely 20, 12 and 4 °C^10^. In addition, degassed champagne at room temperature is used in order to compare the results with the water-ethanol solution (*i*). Finally, a set of experiments will be realized with demineralized water as a reference basis. See “Methods” section for more details.

### Top drop velocity and size

**Figure 4:**
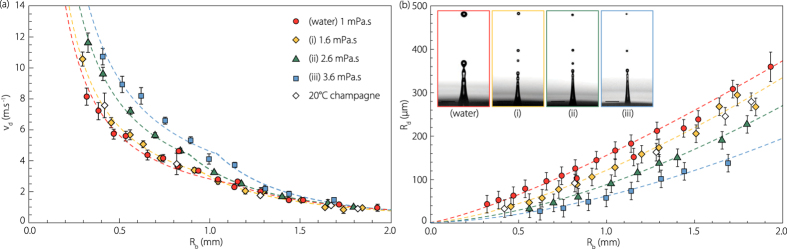
(**a**) Top drop velocity *V*_*d*_ as a function of the bubble radius *R*_*b*_ in water, champagne at 20 °C and in the three hydro-alcoholic solutions (*i*)–(*iii*). The dashed line curves are the best fits of the experimental values of the drop velocity. (**b**) Top drop radius as a function of bubble radius in water, champagne and in the three hydro-alcoholic solutions (*i*)–(*iii*). The dashed lines are the best fits of the four series of data. The four snapshots display the jet and jet drops for each solution, showing that the more viscous the solution, the smaller the top drop.

For the sake of clarity we restrict from now our attention on the droplets that bound the edge of the aerosol cloud. Indeed, we showed^25^ that they highly dominate the evaporation process as they are faster and usually bigger than the others or with a comparable size (Fig. 2). We set out by investigating experimentally in Fig. 4(a) the dependence of the top drop velocity *V*_*d*_ on mother bubble radius *R*_*b*_ in water, champagne and the three hydro-alcoholic solutions (*i*)–(*iii*). *V*_*d*_ is measured when the drop detaches. As expected^17^,^18^,^26^, the drop velocity is greater as the bubble radius is smaller and the liquid is more viscous. Figure 4(b) presents the top drop radius as a function of the bubble radius *R*_*b*_ from our experiments in our five different liquids. The points in water are in perfect agreement with the literature^19^,^27^,^28^. The four snapshots display the jet and jet drops for each solution. The jet is getting thinner and the top drop is shrinking as viscosity is increased. This behaviour, suggested in a previous work^17^, is now clearly quantified. In the two graphs, the dashed lines are the best fit of the experimental drop velocity and radius as a function of the bubble radius. They will be used later on as initial drop radius and velocity in the drop dynamics differential equations. Finally, the superposition of the highest jet drop velocity in champagne and in the water-ethanol solution (*i*) is excellent (see diamonds), making those solutions perfect idealized champagne and confirming that surfactants can be neglected in the jet drop dynamics.

### Maximum drop height

**Figure 5:**
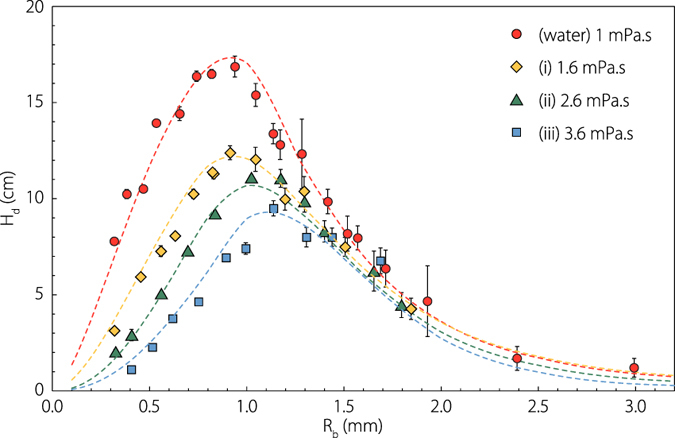
Top drop maximum height as a function of bubble radius in water and for the three hydro-alcoholic solutions (*i*)–(*iii*). The dashed line curves are computed numerically by integrating the Newton’s second law applied to the flying drop using drag coefficient 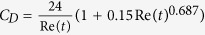, experimental drop radius *R*_*d*_(*R*_*b*_) and initial drop velocity *V*_*d*_(*R*_*b*_) as initial condition.

The top drop maximum height is plotted on Fig. 5 as a function of the bubble radius from our experiments in distilled water and for the three solutions (*i*)–(*iii*). The points in water are in good agreement with the literature^27^,^29^. All these experimental data adopt a bell shape curve with a maximum height reached for bubble radius around one millimeter. The maximum height decreases and is slightly shifted toward larger radius as viscosity is increased. This diminution of the maximum height with viscosity is surprising as we saw that increasing viscosity leads to faster drop ejection velocity. However, because this increase in drop velocity is accompanied by a size shrinking, the effect of drop inertia (which scales with the drop volume), should be compared with drag force F_*D*_ (roughly speaking scaling as a surface). When drop size decreases, surface effects become dominant over volume effects and consequently, small drop inertia becomes negligible compared to the drag force. The maximum height a drop can reach with a given initial velocity can thus be captured by integrating the following differential equation:


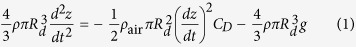


with *z* the drop height and *C*_*D*_ the drop drag coefficient. Here *C*_*D*_ is taken as the drag coefficient on a solid sphere in steady motion. As the drop Reynolds number 
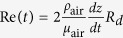
 ranges from ten to a hundred, classical Stokes’ drag 24/Re is not adequate and inertial terms need to be added. Many empirical or semiempirical equations have been proposed^30^ to approximate *C*_*D*_ as a function of the Reynolds number on a given Reynolds range. Here 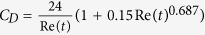 valid for *Re* < 800^31^, has been taken. On Fig. 5 we plot with a colored dashed line the top drop height as a function of the bubble radius computed with this model without any fitting parameters. We observe an excellent agreement between these curves and the experimental points. The drop maximum height is perfectly captured by this simple model containing the experimental drop radius *R*_*d*_(*R*_*b*_) and the initial drop velocity *V*_*d*_(*R*_*b*_) as initial condition. It also confirms the validity of the strong hypotheses of steady drag and drop sphere shape.

### Simple model for drop evaporation

In this system, drop evaporation is crucial to understand the aroma diffusion by champagne aerosol. We have all the informations needed to build a simple predictive model, and to highlight the relevant features playing a role in the evaporation dynamics. Hence, a single droplet with radius *R*_*d*_(*t*), velocity 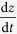 and temperature *T* (between 4 and 20 °C) is moving into an environment with temperature *T*_∞_ = 20 °C and mass fraction of the vapor of the droplet material 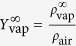. The evaporation process of the droplet depends *a priori* on these parameters. The considered model implies that quasi-steady conditions prevail. Under these conditions, and considering the thermophysical properties as constant, the analysis of mass transfer processes into the gas phase near the droplet surface allows the determination of the regression rate of the droplet radius:


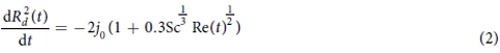


where Sc, the Schmidt number, is defined as the ratio of momentum diffusivity in air (*ν*_air_) and mass diffusivity of vapor in air (*D*), and *j*_0_ the evaporation parameter^32^,^33^. In our case, where the gas temperature is low, evaporation process is only controlled by diffusion, which leads to 
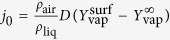
34 - the Stefan flow being negligible^33^. For the calculation of the vapor mass fraction of each substance at the surface of the droplet 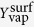, the heat transfer equation and the Clausius-Clapeyron equation are considered. Because the vapor pressure of the glycerol is about six orders of magnitude lower than this of water or ethanol, evaporation of glycerol has been neglected. With the intrinsic difference of volatility between water and ethanol, and an ambient partial pressure taken to 50% of vapor pressure for water (relative humidity) and 0% for ethanol, we obtain an evaporation parameter (*j*_0_) for ethanol around five times greater than evaporation parameter for water. Expectedly, ethanol evaporates easier than water. More details on this analysis are given in the “Methods” section. Equation (2) is the product of two terms: the well known *d*^2^-law for evaporation of an unmoving droplet which follows from equation 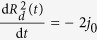
35,36. And, the drop motion which is taken into account using the standard Ranz and Marshall empirical mass transfer correlations for moving sphere 
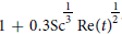
37. This correction comes from the assumption that the mass exchange between the droplet surface and the gas may be modeled as occurring within a spherical diffusion film of constant thickness 
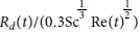
 - this behavior goes by the names of Prandlt-Blasius-Pohlhausen (1921)^38^.

### Drop evaporation from trajectory

**Figure 6:**
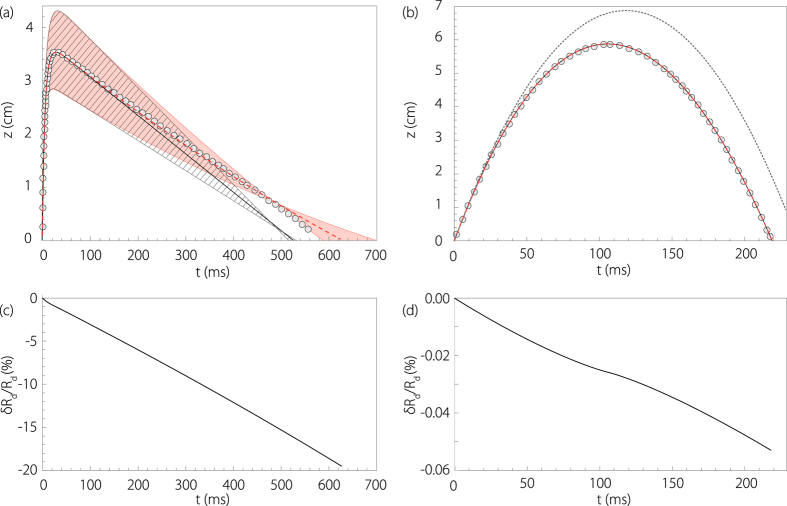
(**a**) Drop height *z* as a function of time for a typical small droplet: *R*_*d*_ = 24 *μ*m and *V*_*d*_ = 8.30 m.s^−1^. The gray circles correspond to the experimental trajectory, the plain black curve corresponds to the trajectory obtained by solving numerically Eq. (1) (no evaporation, *R*_*d*_ constant) and the dashed red curve corresponds to the trajectory obtained by solving numerically Eqs (1) and (2) (with evaporation, *R*_*d*_(t) non constant). The hatched gray and red zones represent the error in the numerical resolution of the respective models (1) and (1) & (2) induced by the experimental error on the initial drop size measurement: *R*_*d*_ = 24 ± 3 *μ*m (experimental error on the initial velocity is negligible). (**b**) Drop height *z* as a function of time for a typical big drop: *R*_*d*_ = 307 *μ*m and *V*_*d*_ = 1.16 m.s^−1^. In this case the experimental trajectory (gray circles) and the numerical trajectory with evaporation (dashed red) and without evaporation (plain black) all collapse. The dotted curve corresponds to the ballistic trajectory 
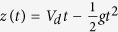
. (**c**) Drop radius variation rate (*δR*_*d*_(*t*)/*R*_*d*_(0), with *δR*_*d*_(*t*) = *R*_*d*_(0) − *R*_*d*_(*t*)) for the drop corresponding to frame (a) (*R*_*d*_(0) = 24 *μ*m), on its time of flight. Here *δR*_*d*_(*t*)/*R*_*d*_(0) reaches a final value of about 20% before landing again. (**d**) Drop radius variation rate for the drop corresponding to frame (b) (*R*_*d*_(0) = 307 *μ*m), on its time of flight. Here *δR*_*d*_(*t*)/*R*_*d*_(0) reaches a final value less than 0.06% before landing again.

Now, the system of differential equations (Eqs (1) and (2)) can be solved, with the variable *R*_*d*_(t) instead of the constant *R*_*d*_ in Eq. (1). The initial conditions 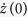 and *R*_*d*_(0) are given by the experimental measurement of the initial drop velocity and radius, *resp. V*_*d*_ and *R*_*d*_ (Fig. 4). On the Fig. 6(a) the experimental height of the first jet drop ejected after a bubble burst is plotted as a function of time with grey circles in the case of a small drop. As expected, the curve is typical of a trajectory where drag forces prevail over inertia. On the same graph, the theoretical trajectories, without evaporation (Eq. (1)), and with evaporation (Eqs (1) and (2)) are plotted respectively with plain black and dashed red curves. Because the determination of the initial radius *R*_*d*_ is not very accurate (*R*_*d*_ = 24 ± 3 *μ*m), the error bar needs to be included in the model. In this case, the hatched gray zone and the red zone represent the error bar in the numerical resolution induced by the experimental error on the initial drop size measurement. Despite the errors and approximations, the experimental trajectory is very well captured by our evaporation model and could not be captured with just a constant radius trajectory model (Eq. (1)). Our evaporation model is therefore well adapted to this system and will be used in the following in order to estimate the evaporated liquid mass during the drop time of flight. The discrepancy at the end of the trajectory may be due to an experimental gradient of humidity getting closer to 100% as *z* gets to 0 (close to the free surface).

Figure 6(c) presents the radius variation (*δR*_*d*_/*R*_*d*_(0)) of the same drop as in Fig. 6(a) on its time of flight. We observe an almost constant radius shrinking rate during the drop free fall, reaching a final value of about 20% before landing again. Obviously, small droplets trajectory are more prone to being affected by evaporation. This is why, on Fig. 6(b), the experimental trajectory of a drop among our biggest (*R*_*d*_ = 307 *μ*m), collapses with the two theoretical trajectories. Evaporation does not modify its trajectory. Indeed, in this case *δR*_*d*_/*R*_*d*_(0) reaches less than 0.06% as shown on Fig. 6(d). Note that, the “plateau” observed on the drop shrinking rate, corresponds to the time when the drop stopped at its maximum height, showing the influence of the drop motion on its evaporation. Finally, on Fig. 6(b) the purely ballistic trajectory without drag has been added with a grey dotted line. We observe that, as the drop size increases, the influence of the drag becomes less significant and the trajectory approximates to a parabola.

## Discussion

**Figure 7:**
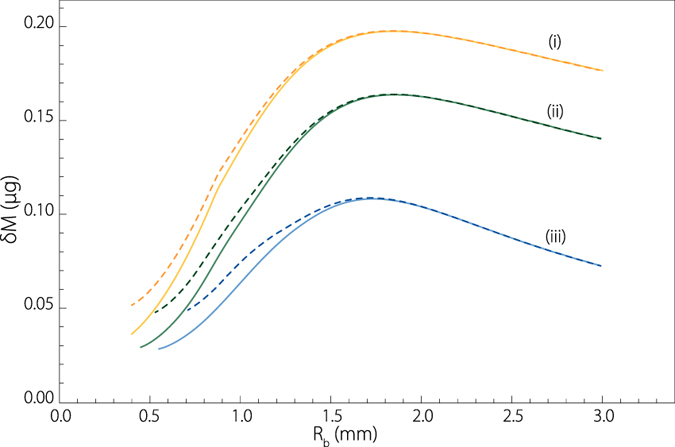
The plain lines present the total evaporated mass *δ*M from the top drop during its time of flight 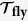, computed numerically with Eqs (1) and (2), as a function of the bubble radius *R*_*b*_ in the three solutions (*i*)–(*iii* ). For the calculation, ambient relative humidity above the tank has been taken to 50%. The dashed lines present the approximated evaporated mass Eq. (3) in the case where 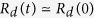 (initial drop radius) with a parabolic trajectory. The approximation is satisfactory for a high part of the range of bubble radius.

Figure 7 presents the bubble radius dependence of the total mass evaporated on the top drop trajectory. Namely, 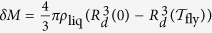 with 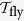 the drop time of flight. The only differences in the calculation of the three curves (*i*)–(*iii*) are the initial conditions 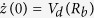 and *R*_*d*_(0) = *R*_*d*_(*R*_*b*_) that change because of viscosity (see Fig. 4). The first interesting result is the absolute value of these curves, around 0.1 *μ*g per drop. Considering that approximately between 300 and 500 bubbles burst per second at the surface of a champagne glass^10^, one obtains an approximative value of the top drops evaporation rate: 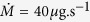. This value needs to be compared to the evaporation rate from the flat surface 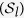 of a glass filled with the same solution. In this aim, the evaporation flux from the surface is taken as purely diffusive and integrated on the length scale of the vapor concentration gradient 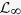
39. This gives the evaporation rate from the surface: 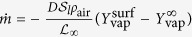. In this context 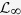 is typically the height between the liquid surface and the top of the glass. In a flute the interfacial area 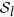 is a disc of approximate radius two centimeters and 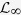 is taken equal to the flute diameter: four centimeters. The other values are the same as for the drop evaporation. In this case, 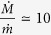, which means that the aerosol constituted by the top drops evaporate ten times more than the still liquid surface. This key result confirms, for the first time, the universal feeling that the characteristic fizz of a sparkling wine is of a paramount importance in the flavor release. Now, let us look whether evaporation of this aerosol can be optimized.

On Fig. 7 each curve has a bell shape, which means that there is a bubble radius optimizing the top drop evaporation. The liquid viscosity also plays a role, the evaporation of the top drop is more efficient if the bubble bursting takes place in a weakly viscous liquid. One observes that, the maximums occur roughly for the same bubble radius. Note that this latter does not correspond either to the maximum drop height, the maximum drop velocity (smallest bubbles) or the maximum drop radius (largest bubbles). However, these particular bubbles (with *R*_*b*_ ~ 1.7–1.8 mm) which would optimize the aroma diffusion in the context of champagne tasting, are quite large. In the popular mind, it is nevertheless often said that the smaller the bubbles, the better the champagne; our results undermine this popular belief, at least in terms of sense of smell. Especially considering that, both the size of bubbles and their nucleation rate are mainly driven by the level of dissolved CO_2_^40^. Indeed, this means that champagnes poor in dissolved CO_2_ (typically old ones) will combine small bubbles and low nucleation rate inducing systematically less numerous bubbles. On the other hand, champagne rich in dissolved CO_2_, combining large bubbles and high bubble nucleation rates will show a much better efficiency in terms of flavor release.

In order to interpret these curves we need to identify the relevant ingredients that influence the evaporation of these jet droplets. To this end, let us consider the limit case where the drop is big enough to allow the trajectory to be approximated by a parabola: 
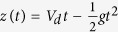
 and 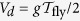. In this case, evaporation does not significantly affect the drop radius: 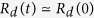 (inset of Fig. 6). Eq. (2) can easily be integrated and the total evaporated mass can be approximated by:


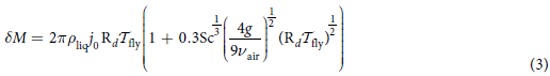


This approximated development of the evaporated mass is plotted on Fig. 7 with dashed line. For the smaller drops, Eq. (3) slightly overestimates the evaporated mass, because in this case the drop trajectory is far from being parabolic and a drop can significantly shrink during its time of flight. However, the agreement is perfect for the bigger drops, including the maximum, particularly well captured. In first approximation, the trajectory of a drop ejected by a bursting bubble is thus relatively well approximated by a parabola. This allows us to identify the product 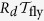 as the relevant quantity for drop evaporation in this system. The first term comes from the evaporation of an unmoving drop of radius *R*_*d*_ in air during the time 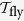. The second term comes from the drop motion. The two terms have the same order of magnitude. *R*_*d*_ and 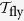 contain all the ingredients needed to estimate the first jet drop evaporated mass.

## Conclusion

This paper presents unique contributions on the bursting bubble aerosol above hydro-alcoholic solutions like champagne or any sparkling wine. First of all, we show experimentally that no film drops populate such aerosol. It is therefore very different from sea spray where film drops are crucial, and it represents an interesting model aerosol only constituted by jet drops. Then, we prove that hydro-alcoholic solutions perfectly mimic champagne in terms of aerosol production. This makes the role of the few surfactants present in champagne negligible in the drop dynamics. Furthermore, we demonstrate that this aerosol plays a critical role in the aroma diffusion. Indeed, compared to a still wine, it drastically improves the transfer of liquid into the surrounding air, even though it is only populated by jet drops. Finally, we exhibit conditions on bubble bursting that optimize aerosol evaporation: large bubbles and weakly viscous liquids. We identify a large bubble radius (~1.8 mm), broadly common to the whole range of champagne viscosity, that makes liquid transfer more efficient. This could be easily achievable as laser-etching on champagne glasses allows the creation of monodisperse bubbles reaching the surface at a chosen radius^41^. This result is also remarkable as it undermines the popular belief that the smaller the bubbles, the better the champagne. Small bubbles being the worst in terms of aroma release. We also show that decreasing champagne viscosity would improve drop evaporation. In this aim, additives that would change wine viscosity without changing the taste might be used. These results pave therefore the way towards the fine tuning of champagne aroma diffusion, a major issue for the sparkling wine industry, and should also encourage further research on this subject.

## Methods

### Experiments

Our experiment consists in releasing a gas bubble from a submerged needle in a liquid and recording the droplets rising above the free surface after the bubble bursts. Bubbles are quasi-steadily formed using a syringe pump and detachment frequency is weak enough to avoid successive bubbles interaction. Different needle diameters (5 < Φ (*μ*m) < 1800) allow us to create bubbles with various radii (*R*_*b*_) ranging from 300 *μ*m to 5 mm. This includes most of the range of bubble radii at the surface of a champagne glass: 200 *μ*m to 1.5 mm^10^. For each bubble size the data presented in this paper represent an average value of a dozen bubble bursting experiments. The bubble collapse, drop velocity and size are analyzed through extreme close-up ultra-fast imagery. Macro lenses and extension rings allow us to record with a definition reaching 5 *μ*m per pixel. Images are obtained between 10000 and 150000 frames per second using a digital high-speed camera. The top drop height is measured using a second digital high-speed camera, triggered by the first camera when the bubble collapses, and with a much greater field of view enabling to follow the drop all along its trajectory with 1000 frames per second. The liquids used in this study include degassed champagne at 20 °C (room temperature), demineralized water and three hydro-alcoholic solutions of surface tension *γ*, viscosity *μ*, density *ρ* with A% of water, B% of ethanol and C% of glycerol in mass (see Table 1). These three solutions enable to reproduce the liquid properties of a usual champagne at three different temperatures between 20 and 4 °C.

### Model

In this model the gas phase heat and mass transfer is considered as quasi-steady, the droplet temperature is considered uniform but can vary with time and the thermophysical properties are treated as a constant^32^,^33^,^34^. There the analysis of heat and mass transfer processes into the gas phase near the droplet surface allows the determination of the instantaneous vaporization rate, 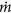. For the mass transfer, this gives 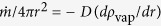 with *D* the mass diffusivity of vapor in air and *ρ*_vap_ the vapor density. By integrating this on the thickness of the spherical diffusion film 
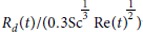
37,38 we obtain an expression of the instantaneous vaporization rate for the moving drop. Because the variation of the liquid temperature is weak, *ρ*_liq_ stays constant and the instantaneous vaporization rate 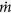 is proportional to the regression rate of the droplet surface: 
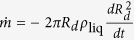
. This finally leads to Eq. (2).

Then, because the Lewis number (Le) defined as the ratio of thermal diffusivity to mass diffusivity is close to unity for water or hydro-alcoholic solution, the analysis of the heat transfer processes into the gas phase at the droplet surface, enables to determine the gas temperature 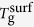 as a function of the vapor mass fraction 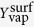, itself dependent on the vapor pressure 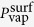. These three parameters at the drop surface can thus be determined with the three following coupled equations:


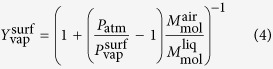


where *P*_atm_ is the pressure near the surface droplet, very close to the the atmospheric pressure. 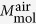 et 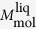 are molar mass of air and liquid.


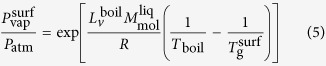


the Clausius-Clapeyron equation, where 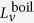 is the latent heat of vaporization, *T*_boil_ the corresponding temperature, and R the gas constant.


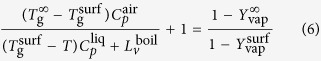


where 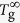 and 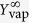 are the temperature and mass fraction far from the droplet, 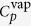 and 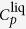 the vapor and liquid specific heat capacity and *T* the liquid temperature taken as uniform in the droplet. By solving these equations, with an ambient partial pressure taken to 50% of vapor pressure for water (relative humidity) and 0% for ethanol, we obtain the following values of the vapor mass fraction of each substance at the surface of the droplet: 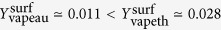. Consequently, we obtain an evaporation parameter (*j*_0_) for ethanol around five times greater than evaporation parameter for water. Expectedly, ethanol evaporates easier than water. Because the vapor pressure of the glycerol is about six orders of magnitude lower than this of water or ethanol, evaporation of glycerol has been neglected. Note that the composition of the solution is kept constant on the drop time of flight because, even if ethanol is more volatile than water, its diffusion time in water is short enough to feed the surface. Finally, by changing the liquid temperatures *T* in the range considered here (*T* ∈ [4, 20 °C]) we observe that *T* has almost no influence on the vapor mass fraction at the surface, which is mainly determined by the thermophysical properties of the liquid and the vapor mass fraction far from the droplets 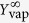. So the assumption of uniform droplet temperature is not restrictive and the liquid temperature variation with time plays no role.

## Additional Information

**How to cite this article**: Ghabache, E. *et al.* Evaporation of droplets in a Champagne wine aerosol. *Sci. Rep.* **6**, 25148; doi: 10.1038/srep25148 (2016).

## Figures and Tables

**Figure 1:**
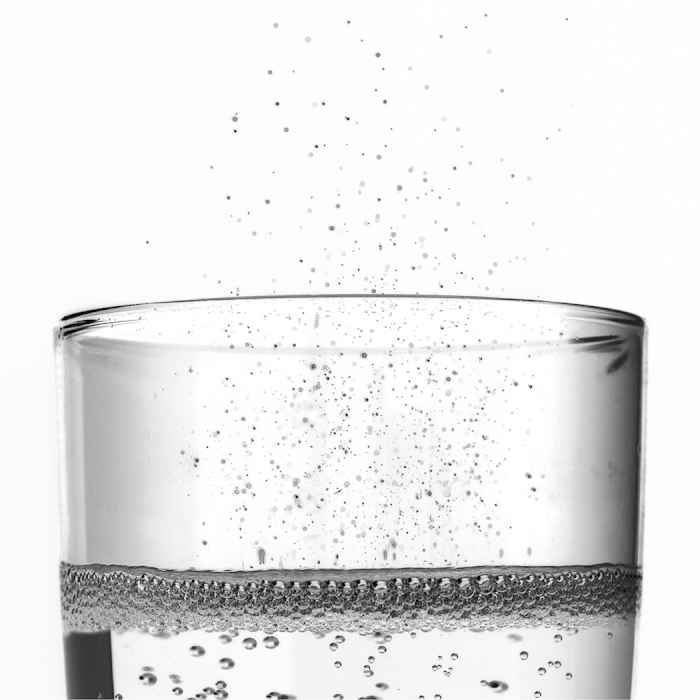
The collapse of hundreds of bubbles at the free surface radiate a cloud of tiny droplets which is characteristic of champagne and other sparkling wines and which complements the sensual experience of the taster (©Alain Cornu/Collection CIVC).

**Table 1:** Characteristics of the five solutions of the study.

Solution	A	B	C	*μ*	*ρ*	*γ*
Water	100	0	0	1.0	1000	72
Champagne	–	–	–	1.6	992	48
(i)	89.5	10.5	0	1.6	983	48
(ii)	66.6	7.6	25.8	2.6	1048	48
(iii)	55.3	4.9	39.8	3.6	1082	48

A, B and C: mass % of water, ethanol and glycerol. *μ*, *ρ* and *γ*: viscosity (mPa.s), density (kg.m^−3^) and surface tension (mN.m^−1^) measured at 20 °C.
